# High Mitochondrial DNA Copy Number and Bioenergetic Function Are Associated with Tumor Invasion of Esophageal Squamous Cell Carcinoma Cell Lines

**DOI:** 10.3390/ijms130911228

**Published:** 2012-09-10

**Authors:** Chen-Sung Lin, Hui-Ting Lee, Shu-Yu Lee, Yao-An Shen, Liang-Shun Wang, Yann-Jang Chen, Yau-Huei Wei

**Affiliations:** 1 Institute of Clinical Medicine, National Yang-Ming University, Taipei 112, Taiwan; E-Mails: doc2765c@ms59.hinet.net (C.-S.L.); htlee1228@gmail.com (H.-T.L.); yjchen@ym.edu.tw (Y.-J.C.); 2 Faculty of Medicine, National Yang-Ming University, Taipei 112, Taiwan; 3 Division of Thoracic Surgery, Department of Surgery, Taipei Hospital, Department of Health, Executive Yuan, New Taipei City 242, Taiwan; 4 Division of Allergy, Immunology and Rheumatology, Department of Internal Medicine, Wan-Fang Hospital, Taipei Medical University, Taipei 116, Taiwan; 5 Department of Biochemistry and Molecular Biology, National Yang-Ming University, Taipei 112, Taiwan; E-Mails: r98442018@ntu.edu.tw (S.-Y.L.); yaoan.shen@gmail.com (Y.-A.S.); 6 Department of Life Science and Institute of Genome Sciences, National Yang-Ming University, Taipei 112, Taiwan; 7 Division of Thoracic Surgery, Department of Surgery, Taipei Medical University—Shuang Ho Hospital, New Taipei 235, Taiwan; E-Mail: wangls72269@yahoo.com.tw; 8 Department of Medicine, Mackay Medical College, New Taipei City 252, Taiwan

**Keywords:** bioenergetic function, esophageal squamous cell carcinoma (ESCC), mitochondrial DNA (mtDNA), invasion, epithelial mesenchymal transition (EMT)

## Abstract

We previously reported a gradual increase of relative mitochondrial DNA (mtDNA) copy number during the progression of esophageal squamous cell carcinoma (ESCC). Because mitochondria are the intracellular organelles responsible for ATP production, we investigated the associations among mtDNA copy number, mitochondrial bioenergetic function, tumor invasion and the expression levels of epithelial mesenchymal transition (EMT) markers in a series of seven ESCC cell lines, including 48T, 81T, 146T, TE1, TE2, TE6 and TE9. Among them, TE1 had the highest relative mtDNA copy number of 240.7%. The mRNA of mtDNA-encoded ND1 gene (2.80), succinate-supported oxygen consumption rate (11.21 nmol/min/10^6^ cells), ATP content (10.7 fmol/cell), and the protein level of mitochondrial transcription factor A (TFAM) were the highest and the lactate concentration in the culture medium (3.34 mM) was the lowest in TE1. These findings indicate that TE1 exhibited the highest bioenergetic function of mitochondria. Furthermore, TE1 showed the highest trans-well migration activity of 223.0 cells/field, the highest vimentin but the lowest E-cadherin protein expression levels, which suggest that TE1 had the highest invasion capability. We then conducted a knockdown study using pLKO.1-based lentiviral particles to infect TE1 cells to suppress the expression of TFAM. Molecular analyses of the parental TE1, control TE1-NT and TFAM knockdown TE1-sh-TFAM(97) cells were performed. Interestingly, as compared to the control TE1-NT, TE1-sh-TFAM(97) exhibited lower levels of the relative mtDNA copy number (*p* = 0.001), mRNA of mtDNA-encoded ND1 gene (*p* = 0.050), succinate-supported oxygen consumption rate (*p* = 0.065), and ATP content (*p* = 0.007), but had a higher lactate concentration in the culture medium (*p* = 0.010) and higher protein level of lactate dehydrogenase. A decline in mitochondrial bioenergetic function was observed in TE1-sh-TFAM(97). Significantly, compared to the control TE1-NT, TE1-sh-TFAM(97) had a lower trans-well migration activity (*p* < 0.001), a higher E-cadherin level but a lower vimentin protein level, which indicates a decrease of invasiveness. Taken together, we suggest that high relative mtDNA copy number and bioenergetic function of mitochondria may confer an advantage for tumor invasion of ESCC.

## 1. Introduction

Esophageal squamous cell carcinoma (ESCC) is an aggressive neoplasm in Asia, especially in Southern China, Hong Kong and Taiwan [1–5]. In a previous study of 72 pairs of ESCC clinical samples, we found that the relative copy number mitochondrial DNA (mtDNA) was increased gradually from the non-cancerous esophageal mucosa to cancerous ESCC nest and then the metastatic lymph node [6]. In light of these findings, and the fact that mitochondria are the intracellular organelles responsible for ATP production to meet the energy demand of human cells [7], we investigated whether mtDNA copy number is related to energy production and plays an important role in the progression of ESCC.

A complete metabolism of one mole of glucose can generate a maximum of 36–38 mol of ATP, including two moles of ATP after glycolysis in the cytoplasm (oxygen independent) and 34–36 moles of ATP from oxidative phosphorylation in the mitochondria (oxygen dependent). During glycolysis, one mole of glucose can be converted into two moles of pyruvate, which then enters the Krebs cycle for subsequent electron transport and oxidative phosphorylation in the mitochondria to generate ATP. Under some circumstances, when oxygen supply is not sufficient or the mitochondria are impaired, pyruvate is transiently reduced to lactate by lactate dehydrogenase (LDH) in cytoplasm without entering the mitochondria. This kind of glucose metabolism is termed anaerobic glycolysis or lactate fermentation, and only two moles of ATP can be generated by incomplete catabolism of one mole of glucose [8].

In contrast to normal human tissues, human cancers can display an avid glucose uptake with profound lactate production to generate ATP, even if the surrounding oxygen supply is sufficient. This special phenomenon is termed Warburg effect, which was first observed by Dr. Otto Warburg eight decades ago. He contended that human cancer tissues, unlike normal tissues, obtain an increase of ATP from lactate fermentation in cytoplasm and a decreased reliance on the ATP generated by oxidative phosphorylation in mitochondria. Such kind of metabolic shift in cancer tissues led Warburg to speculate that the function of respiratory enzyme complexes in cancer tissues might be impaired or suppressed [9–12].

Generally, there is a mitochondrial network within human cells, and each mitochondrion is connected to other mitochondria during most of the time. However, due to the dynamic processes of fission and fusion of this network, many small mitochondria are disintegrated from the main network that can be observed in different locations at certain moments or at different times inside the cells. This is dynamic and in a particular snapshot, some cells may appear to contain several small mitochondria, besides the un-fragmented mitochondrial reticulum. Only under stress or harsh conditions, especially in a pathological state, the mitochondrial reticulum can be disintegrated in fragments. Generally, each fragment contains at least a single nucleoid and each nucleoid harbors around 1 to 6 mtDNA molecules [13]. Thus, it is established that the mtDNA copy number is highly dynamic and varies widely with the cell type and physiological conditions. The tissue cells that have a high energy demand usually have larger amounts of mtDNA [14].

Human mtDNA is a circular and double-stranded DNA with a size of 16,569 bp, including the non-coding and coding regions. The coding region encodes 13 polypeptides that constitute the respiratory enzyme complexes, 2 rRNAs, and a set of 22 tRNAs required for protein synthesis in the mitochondria. The 13 mtDNA-encoded polypeptides include 7 (ND1, ND2, ND3, ND4, ND4L, ND5, and ND6) in Complex I, 1 (cytochrome *b*) in Complex III, 3 (COX I, COX II and COX III) in Complex IV, and 2 (ATPase 6 and ATPase 8) in Complex V [15]. In addition, approximately 90 polypeptides constituting the respiratory enzyme complexes are encoded in nuclear DNA (nDNA). All the subunits of Complex II (succinate-coenzyme Q oxidoreductase) are totally nDNA-encoded. The non-coding region is 1124 bp long and is also called D-loop (displacement loop), which plays an important role in the execution and regulation of replication and transcription of mtDNA [16–18]. Mitochondrial transcriptional factor A (TFAM) can bind to the D-loop and thereby regulate the replication and transcription of mtDNA and is essential for mitochondrial biogenesis [17,19]. Although mitochondria have their own genomes, all the proteins participating in mtDNA replication and transcription are encoded by nDNA [16–18].

During the past decades, biomedical researchers have paid increasing attention to the alterations of relative mtDNA copy number in human cancers. Compared to the corresponding non-cancerous parts, a decrease of the relative mtDNA copy number was found in lung cancer, hepatocellular carcinoma, gastric cancer and breast cancer [20–22]. In contrast, a progressive increase of the relative mtDNA copy number was found in the carcinogenesis of head and neck cancers and in the progression of esophageal squamous cell carcinoma [6,23]. The disparity of these findings in the alteration of relative mtDNA copy number among different cancers deserves further study. In addition, the relationship between relative mtDNA copy number and mitochondrial function in ESCC cell lines remains unclear. In this study, we determined the relative mtDNA copy number and its association with mitochondrial biogenesis and cancer invasiveness among 7 ESCC cell lines, respectively. One of these 7 cell lines, subsequently underwent TFAM knockdown for a further study of the relationships among the relative mtDNA copy number, bioenergetic function and the invasive phenotype of ESCC.

## 2. Results and Discussion

### 2.1. The Distribution of Relative mtDNA Copy Numbers of the 7 ESCC Cell Lines

A total of 7 ESCC cell lines, including CE-48T/VGH (48T), CE-81T/VGH (81T), CE-146T/VGH (146T), TE1, TE2, TE6, and TE9 were used to conduct this study [24,25]. The growth medium was composed of Dulbecco’s modified Eagle’s medium (DMEM) plus 10% fetal bovine serum (FBS), 1% non-essential amino-acids, 1% of L-glutamine and 1% of a mixture of penicillin (10,000 U/mL) and streptomycin (10,000 μg/mL). Concerning the distribution of the relative mtDNA copy number among the 7 cell lines, a significant difference was noted (*p* < 0.001, Table 1), and TE1 had the highest values of 240.7% (when the relative mtDNA copy number of 143B cell was defined as 100%).

### 2.2. Relative mtDNA Copy Number and Mitochondrial Bioenergetic Function of the Seven ESCC Cell Lines

Besides the relative mtDNA copy number, significant differences were also noted in the succinate-supported oxygen consumption rate (*p <* 0.001), the ATP content (*p <* 0.001) and the lactate concentration in the cultured medium (*p* < 0.001) among the 7 ESCC cell lines. Interestingly, TE1 also had the highest levels of succinate-supported oxygen consumption rate of 11.21 nmol/min/10^6^ cells, and the ATP content of 10.67 fmol/cell, respectively. However, TE1 had the lowest lactate concentration of 3.34 mM in the cultured medium (Table 1).

### 2.3. Expression Levels of Proteins and mtDNA Encoded mRNAs Related to Mitochondrial Biogenetic Function among the Seven ESCC Cell Lines

**Figure 1:**
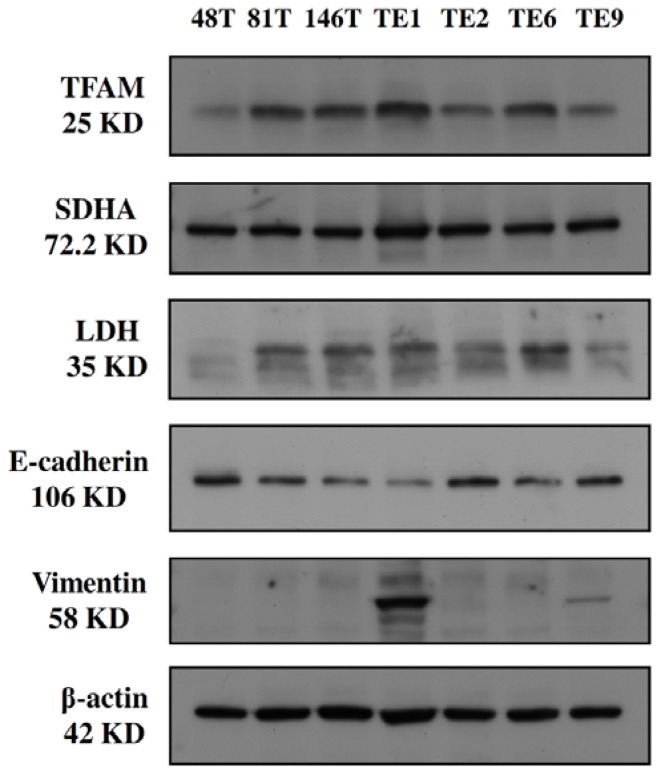
Western blot analysis shows that TE1 had the highest mitochondrial transcription factor A (TFAM) (**the first row**); relative higher succinate dehydrogenase A (SDHA) (**the second row**); medium lactate dehydrogenase (LDH) (**the third row**); lowest E-cadherin (**the fourth row**) and highest vimentin (**the fifth row**) protein expression. The expression of beta-actin (**the sixth row**) was used as an internal control.

Regarding the protein expression, as shown in Figure 1, TE1 had the highest protein level of TFAM, higher expression of SDHA (succinate dehydrogenase A, a subunit of respiratory enzyme Complex II) and medium-level of expression of LDH among the 7 cell lines. Furthermore, among the 7 ESCC cell lines, an obvious difference in the relative mRNA expression of the mtDNA-encoded ND1 gene was noted (*p* < 0.001, Table 1). In addition, TE1 had the highest mRNA level of 2.80 when the relative ND1 mRNA expression of 143B cell was defined as 1.00.

Most studies have revealed that a high relative mtDNA copy number is associated with a high mitochondrial bioenergetic function in human cells [14,26,27]. In agreement with this, among the 7 ESCC cell lines we examined TE1 had the highest relative mtDNA copy number and bioenergetic function, including the highest oxygen consumption rate, highest ATP content but the lowest lactate concentration in the culture medium. Since TFAM plays an important role in the regulation of mitochondrial biogenesis [17,19], it is reasonable to find that TE1 had the highest TFAM protein level, which led to the highest relative mtDNA copy number and mRNA level of mtDNA-encoded *ND1* gene. The above findings imply that a high relative mtDNA copy number is related to a high mitochondrial bioenergetic function among the 7 ESCC cell lines.

It is well established that only 13 polypeptides of the respiratory enzyme complexes are encoded by mtDNA, and all the other peptides are encoded by nDNA. Because the 4 polypeptides of Complex II are totally encoded by nDNA, our finding that TE1 had a relative higher protein expression of SDHA might be caused by the compensatory gene induction. As a result, the high mitochondrial bioenergetic function of TE1 cells might be attributed, at least in part, by the nDNA-encoded polypeptides.

Although TE1 showed the highest bioenergetic function and lowest lactate concentration in the cultured medium, TE1 did not express the lowest level of LDH but a medium one. LDH can convert pyruvate to lactate in cytoplasm to guarantee the execution of glycolysis [28]. This interesting finding indicates that TE1 had the abilities to metabolize pyruvate through either mitochondrial respiration or lactate fermentation to generate ATP, which is quite compatible with Warburg effect [9–12]. Another pathway might be turned on to deal with the intracellular lactate, because TE1 had the lowest lactate concentration in the cultured medium. Actually, LDH is a tetrameric enzyme comprising two major subunits A and/or B, resulting in five isozymes (A4, A3B1, A2B2, A1B3, and B4) that can catalyze the forward and backward conversion of pyruvate to lactate. LDHA (LDH-5, MLDH, or A4), which is the predominant form in skeletal muscle, mainly converts pyruvate to lactate. LDHB (LDH-1, H-LDH, or B4), which is found in heart muscle, mainly converts lactate to pyruvate. It has long been known that the LDHA levels of many human cancer tissues are higher than those of normal tissues [29]. The primary antibody used in this study is a polyclonal antibody and it could not differentiate specific forms of LDH. However, the results provided useful information for better understanding of metabolic alteration in ESCC cell lines. Clinically, the expression of LDH, mainly the LDH-5 (or LDH-A4), had been evaluated in colon cancer, endometrial cancer and lung cancer, and its high expression was found to be related to a poor prognosis [30–32].

### 2.4. Trans-Well Migration Activity, Expression of Epithelial-Mesenchymal Transition (EMT) Markers and Growth Kinetics of the 7 ESCC Cell Lines

**Figure 2:**
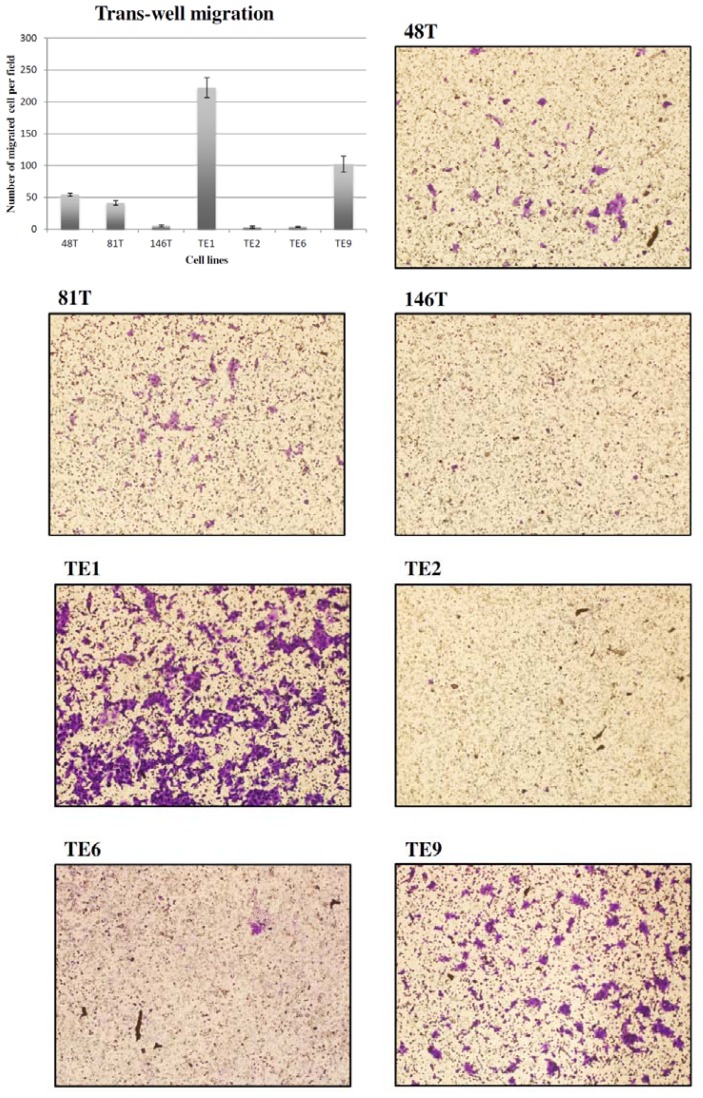
Illustration of the quantitative (bar, cells/field) and photographic (light microscopy) results of the assay of trans-well migration activity among the seven cell lines. TE1 exhibited the highest trans-well migration activity.

**Figure 3:**
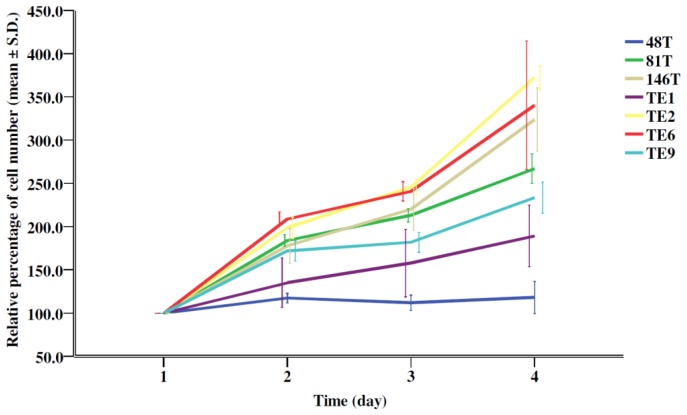
Growth kinetics of the seven ESCC cell lines. Data are shown in percentage when the cell number on day 1 is defined as 100.0%. The graph was plotted by using the SPSS 12.0 software (SPSS Inc, Chicago, IL, USA).

As shown in Table 1 and Figure 2, a significant difference in the trans-well migration activity was noted among the 7 ESCC cell lines, and TE1 exhibited the highest trans-well migration activity of 223 cells/field. Regarding the expression of EMT markers (Figure 1), TE1 had the highest expression levels of vimentin (a mesenchymal cell marker) but the lowest E-cadherin (an epithelial cell marker). Concerning the growth kinetics (Figure 3), TE1 showed the second to the last in the growth rate.

Now that TE1 had the highest relative mtDNA copy number and highest mitochondrial bioenergetic function with the highest intracellular ATP content, we were interested to know whether TE1 exhibited a higher migration activity. Significantly, TE1 did express the highest trans-well migration activity. To avoid the confounding effect of cell proliferation to interfere with the analysis of trans-well migration activity, the growth kinetics of the 7 cell lines were also evaluated, and the results showed that the growth rate of TE1 was second to the last. This finding convinced us that there must be a relationship between the bioenergetic function of mitochondria and the migration activity of the ESCC cell line. It is well established that a decrease in epithelial cell marker and an increase in mesenchymal cell marker, the EMT, play an important role in the invasive process of cancers [33]. We found that TE1 expressed the lowest E-cadherin and the highest vimentin levels. The results are compatible with the observation that TE1 had the highest trans-well migration activity.

Based on the above findings of the 7 ESCC cell lines, we hypothesized that higher relative mtDNA copy number is associated with a higher mitochondrial bioenergetic function and a higher invasiveness of the ESCC cell line. However, such an association needs to be further validated. Thus, we conducted the following gene knockdown experiments.

### 2.5. Knockdown Efficiency of TFAM and Its Effect on Growth Kinetics of ESCC

**Figure 4:**
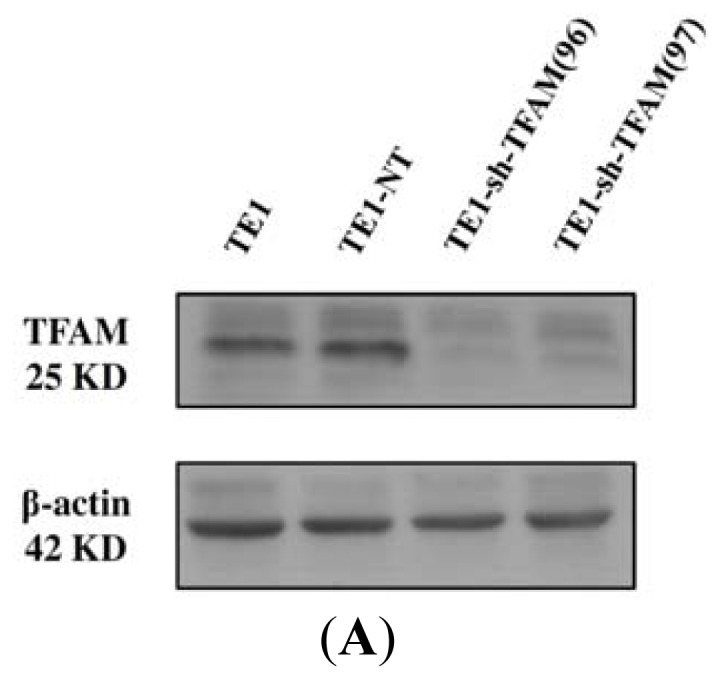

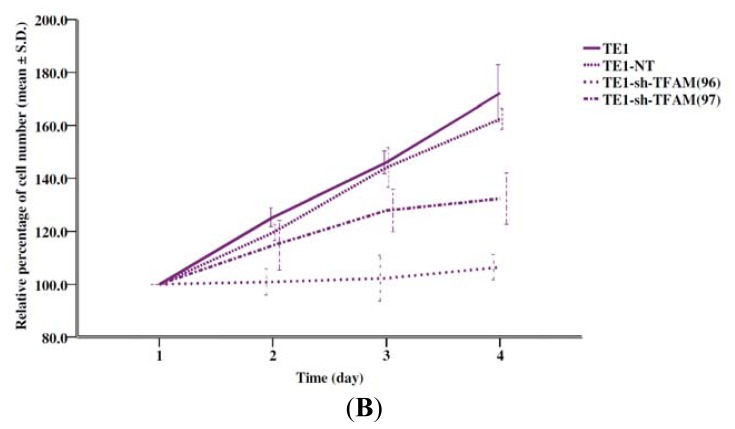
(**A**) Western blot analysis of the TFAM expression among the parental TE1, control TE1-NT, and TFAM knockdown TE1-sh-TFAM(96) and TE1-sh-TFA(97) cells; (**B**) The growth kinetic curves of the parental TE1, control TE1-NT, and TFAM knockdown TE1-sh-TFAM(96) and TE1-sh-TFA(97) cells, respectively, were plotted by SPSS 12.0 software (SPSS Inc, Chicago, Ill). Data are shown in percentage when the cell number of each cell line on day 1 was defined as 100.0%.

As shown in Figure 4A, the TFMA expression levels of parental TE1, control TE1-NT, and TFAM knockdown TE1-sh-TFAM(96) and TE1-sh-TFAM(97) were examined. There was no obvious difference in the TFAM expression between the parental TE1 and the control TE1-NT cells. However, a significant knock-down efficiency was achieved in TE1-sh-TFAM(96) and TE1-sh-TFAM(97) cells, with a higher knockdown efficiency in TE1-sh-TFAM(96) cells. As shown in Figure 4B, there was an obvious growth arrest in TE1-sh-TFAM(96) cells, and no significant difference was noted between the parental TE1 and the control TE1-NT cells. Thus, we only focused on parental TE1, control TE1-NT and TE1-sh-TFAM(97) cells in the following experiments.

### 2.6. Comparisons of Mitochondrial Bioenergetic Function among Parental TE1, Control TE1-NT and TFAM Knockdown TE1-sh-TFAM(97) Cells

**Figure 5:**
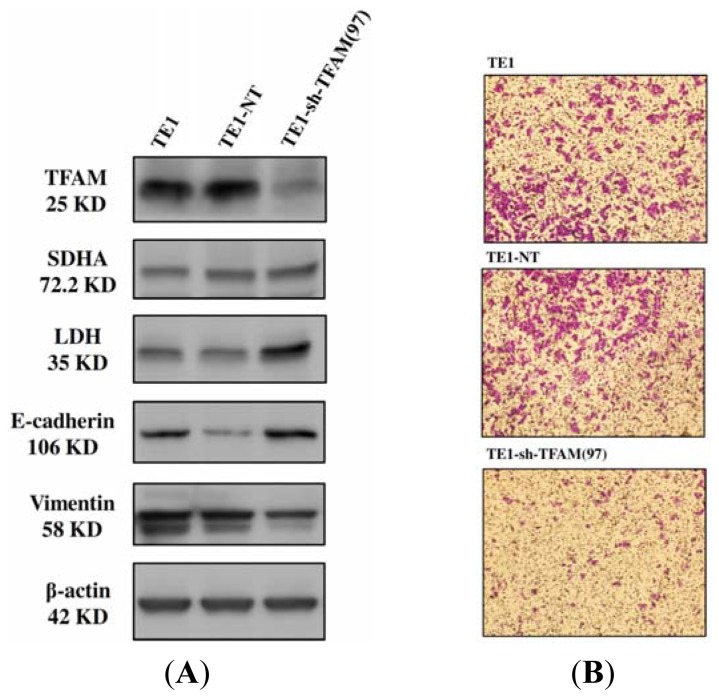
(**A**) Western blot analysis showed that the TFAM knockdown TE1-sh-TFAM(97) had the lowest TFAM (the first row), equal SDHA (the second row), highest LDH (the third row), highest E-cadherin (the fourth row) and lowest vimentin (the fifth row) protein expressions. The expression of beta-actin (the sixth row) was used as an internal control; (**B**) Illustrations are photographic (light microscopy) results of the trans-well migration activity among the parental TE1 cell, control TE1-NT cell, and TFAM knockdown TE1-sh-TFAM (97) cells. TE1-sh-TFAM(97) exhibited the lowest trans-well migration activity.

As shown in Table 2, the relative mtDNA copy number of TE1-sh-TFAM(97) was significantly lower than that of control TE1-NT (152.5% *vs*. 227.8%, *p* = 0.001) after the knockdown of *TFAM*. TE1-sh-TFAM(97) also exhibited a lower relative mRNA level of ND1(*p* = 0.050), a lower succinate-supported oxygen consumption rate (*p* = 0.065) and a lower intracellular level of ATP (*p* = 0.007) than those of the control TE1-NT. However, there were no significant differences between the parental TE1 and control TE1-NT among the above parameters. The mitochondrial bioenergetic function was significantly down-regulated in TE1-sh-TFAM(97). Because only 13 polypeptides of the respiratory enzyme complexes are encoded by mtDNA, it is considered that the knockdown of TFAM would not interfere with the expression of nDNA-encoded polypeptides of respiratory enzyme complexes. As illustrated in Figure 5A, there was no obvious difference in the SDHA expression among the parental TE1, control TE1-NT and the TFAM knockdown TE1-sh-TFAM(97) cells. This finding confirmed that the impaired mitochondrial bioenergetic function of TE1-sh-TFAM(97) cells was the consequence of the decrease in the mtDNA copy number and mtDNA-encoded polypeptides after the knockdown of TFAM.

### 2.7. Alterations in Metabolism, Trans-Well Migration and Expression of Epithelial–Mesenchymal Transition Markers in Parental TE1, Control TE1-NT and TFAM Knockdown TE1-sh-TFAM(97) Cells

As shown in Table 2 and Figure 5A, TE1-sh-TFAM(97) exhibited a higher LDH expression and a higher concentration of lactate in the cultured medium (*p* = 0.010) than those of control TE1-NT cells. Moreover, TE1-sh-TFAM(97) showed an increase in the expression of E-cadherin, a decrease in the expression of vimentin and a decrease in the trans-well migration activity (*p* < 0.001) as compared with those of control TE1-NT cells

The above findings in TE1 cells were consistent with the Warburg effect, because the impaired mitochondria could be compensated for by an increase of lactate fermentation to keep up the glycolysis to generate ATP. However, the ATP from the glycolysis seems not enough to make up the ATP from the mitochondrial respiration, because the ATP production of TE1-sh-TFAM(97) was less than that of control TE1-NT. Thus, the growth rate of TE1-sh-TFAM(97) was slightly slower than that of control TE1-NT, but was not significantly arrested. Moreover, the trans-well activity of the TE1-sh-TFAM(97) was significantly decreased when compared to the control TE1-NT cells, and this alteration was much more obvious than the change of growth kinetics. An elevated expression of E-cadherin and decreased expression of vimentin in TE1-sh-TFAM(97) were correlated well with its decrease in trans-well migration activity. These knockdown results further proved our hypothesis that mtDNA copy number is associated with mitochondrial bioenergetic function and invasiveness of ESCC.

When evaluating the relationship between mitochondrial bioenergetic function and cancer, most researchers paid their attention to the Warburg effect and emphasized the mitochondria impairment in cancers [34,35]. However, the key concept that Dr. Warburg conveyed is that cancer cells can obtain an increased amount of ATP from fermentation besides from mitochondrial respiration, whereas the normal body cells obtain much more energy from respiration than from fermentation [9–11,36]. Depending on the surrounding environment, cancer cells can generate ATP through either mitochondrial respiration or lactate fermentation, and the dual abilities confer them higher aggressive and invasive activities [34–37]. In a study of the tumors of lung cancer patients, if cancer tissues used both mitochondrial respiration and lactate fermentation, the patients had the worst prognosis [32]. Similarly, TE1 had the highest intracellular ATP level and the dual abilities to metabolize pyruvate through either mitochondrial respiration or lactate fermentation to generate ATP and such characteristics rendered TE1 cell the highest bioenergetic function and invasion activity. Although one of the dual activity of TE1 was suppressed by the knockdown to TFAM to block the mitochondrial respiration, the *TFAM* knockdown TE1-sh-TFAM(97) cells still had the ability to grow and to do the trans-well migration but at a lower level. This explains that, unlike normal human cells, cancer cells can survive a persistent hypoxia condition using anaerobic glycolysis and further validated the Warburg effect.

Regarding the Warburg effect in human cancers, most researchers emphasized the increase in the uptake of glucose to produce ATP. It deserves to mention that when we cultured ESCC, besides the original L-glutamine in the DMEM, an additional 1% L-glutamine was added to the culture medium. Obvious growth retardation occurred if the extra L-glutamine was not added. Since the 1950s, several cancer biologists also noted the importance of L-glutamine as a tumor nutrient. Several novel roles of L-glutamine in cancer biology have been identified, including the essential role of the amino donor in purine biosynthesis, and supply of alpha-keto glutarate to the Krebs cycle and involvement in the production of other macromolecules [38]. However, we did not examine the role of glutamine and its relationship to glucose metabolism in this study. It is established that under the help of glutaminase in mitochondria, glutamine can be converted to alpha-keto glutarate and then enters the Krebs cycle for subsequent oxidative metabolism. In our knockdown study, the relative mtDNA copy number and mtDNA-encoded polypeptides were significantly reduced to cause a suppression of respiratory enzyme complexes. As a result, oxidative metabolism of both glucose and L-glutamine in mitochondria was inhibited and contributed to the impairment of bioenergetic function and decreased trans-well activity.

## 3. Experimental Section

### 3.1. Extraction of Total Cellular DNA, DNA Free RNA and Protein

DNA extraction. Approximately 3 × 10^6^ cells were mixed with 500 μL of Tris-EDTA buffer, 50 μL of 10% sodium dodecyl sulfate and 10 μL of proteinase K (20 mg/mL) and incubated at 56 °C for 16 h. Subsequent phenol, phenol/chloroform and chloroform (500 μL) extractions were performed to get the supernatants in three steps and then the DNA was salted out by the addition of isopropanol (500 μL) plus sodium acetate (50 μL) (3 M, pH 5.0) and kept at −20 °C for 16 h. After centrifugation at 12,000*g* at 4 °C for 30 min and being washed with 75% cooled alcohol solution, the DNA pellet was dried by vacuum and then dissolved in distilled water and kept at −20 °C until use [39].

RNA extraction. Approximately 3 × 10^6^ cells were mixed with 500 μL of TRI™ Reagent (Sigma-Aldrich Chemical Co.) plus 100 μL of chloroform to centrifugation at 12,000*g* at 4 °C for 15 min. The supernatant containing RNA was precipitated by the addition of 250 μL of isopropanol. After centrifugation at 12,000*g* at 4 °C for 10 min and being washed with 75% alcohol for 2 times, the RNA pellet was dried at 4 °C for 30 min and then dissolved in distilled water, which contained 0.1% of diethylpyrocarbonate (DEPC). A total of 10 μg RNA was further purified in a reaction containing DNase to discard the residual DNA, because the mtDNA structure harbors no intron. Finally, a total of 2 μg of purified DNA-free RNA was reversed-transcribed to cDNA with the Ready-to-go RT-PCR kit (GE Healthcare UK) by oligo-dT primers [40].

Protein extraction. Approximately 3 × 10^6^ cells were suspended in 50 μL of lysis buffer (50 mM Tris-HCl, 0.25% sodium deoxycholate, 150 mM NaCl, 1 mM EDTA, 1% Triton X-100, and 1% NP-40, pH 7.4) containing 1% of protease inhibitor (Roche Applied Sciences). The suspension was incubated at 4 °C for 30 min and then centrifuged at 12,000*g* for 20 min at 4 °C. The supernatant that contained total cellular protein was collected and kept at −80 °C until use.

### 3.2. Determination of the Relative mtDNA Copy Number, mtDNA-Encoded mRNA and Protein Expression Levels

Quantitative real-time PCR (Q-PCR) using SYBR Green I (Roche Applied Science, Mannheim, Germany) to determine the threshold cycle (Ct) was applied for the quantification of DNA and mRNA, which were described previously [6,41–43]. Standard curves represented for primers’ replication efficiency were established by using DNA and cDNA from 143B osteosarcoma cell line.

The mtDNA copy number was defined as total mtDNA copies divided by total nDNA copies. The sequences of primers used for mtDNA amplification (ND1 region) and nDNA amplification (18S rRNA region) were mtF3212: 5′-CACCCAAGAACAGGG TTTGT-3′ and mtR3319: 5′-TGGCCAT GGGTATGTTGTTAA-3′ as well as 18SF1546: 5′-TAGAGGGACAAGTGGCGTTC-3′ and 18SR1650: 5′-CGCTGAGCCAGTCAGTGT-3′, respectively [6,42,44]. The *R^2^* for standard curves was 0.9995 for mtDNA and 0.9996 for nDNA, respectively.

The mtDNA encoded mRNA expression was defined as total ND1 copies divided by total 18S rRNA copies. The sequences of primers used for mtDNA encoded ND1 and nDNA encoded 18S rRNA mRNA were ND1F: 5′-TGGGTACAATGAGGAGTAGG-3′ and ND1R: 5′-GGAGTAATCC AGGTCGGT-3′ as well as 18S rRNAF: 5′-CTCAACACGGGAAACCTCAC-3′ and 18S rRNA R: 5′-CGCTCCACCAACTAAGAACG-3′, respectively [41,43,45]. The *R^2^* for standard curves was 0.9999 for ND1 and 0.9990 for 18S rRNA, respectively.

For each reaction, 1 μL of sample DNA (10 ng/μL)/1 μL of cDNA (16× dilution) was amplified in a 10-μL reaction buffer containing 0.25 μL (20 μM) of each primer (mtF and mtR for mtDNA, 18SF and 18SR for nDNA; ND1F and ND1R for mtDNA-encoded mRNA, 18S rRNAF and 18S rRNAR for nDNA-encoded mRNA), 1.2 μL of 3 mM MgCl_2_, 1 μL of LightCycler SYBR Green I mixed reagent (Roche Applied Science, Mannheim, Germany) and 6.3 μL of PCR grade H_2_O. Simultaneously, 1 μL of DNA (1 ng/μL)/cDNA (16× dilution) from the 143B cells and PCR grade H_2_O were included as the positive and negative controls, respectively. The PCR procedures included hot start at 95 °C (10 min) and 40 cycles of 95 °C (20 s), 62 °C (20 s) and 72 °C (20 s). The fluorescence intensity was measured at the end of primer extension at 72 °C for Ct calculation. A melting curve was detected from 55 °C to 95 °C to confirm the PCR product that we interested in. The relative mtDNA copy number and relative mRNA levels of mtDNA-encoded ND1 of each sample were calculated, after adjusting the mtDNA copy number of 143B cell as 100% and the mRNA level of mtDNA-encoded genes of 143B cell as 1.00 by the standard cures we established. Each reaction was done in duplicate and experiments were repeated for 3 independent cultures of cell lines (*N* = 3). The mean value and standard deviation were calculated for data presentation.

The relative protein expression levels were determined by Western blot. An aliquot of 60 μg of total cellular protein was separated on a 10% SDS-PAGE, and then blotted onto a piece of BioTrace™ polyvinylidene difluoride (PVDF) membrane (Pall Corp: Pensacola, FL, USA). Non-specific bindings were blocked with HyCell blocking buffer (HyCell Biotechnology Inc.: South Logan, UT, USA) for 1 h at room temperature. The membrane was subjected to specific primary antibodies against mtTFA (Santa Cruz, sc-19050, 1:500, 25 kD), SDHA (Molecular Probes, A11142, 1:1000, 72.2 kD), lactate dehydrogenase (LDH) (Santa Cruz, sc-33781, 1:5000, 35 kD), E-cadherin (Upstate, 07-697, 1:1000, 106 kD), vimentin (Sigma, V6630, 1:2000, 58 kD) and beta-actin (Chemicon, MAB1501, 1:10,000, 42 kD), and incubated for 16 h at 4 °C. After 4 times of wash by PBST buffers, the membrane was incubated with horseradish peroxidase (HRP)-conjugated secondary antibodies for 1 h at room temperature, the protein band was visualized on an X-ray film (Fujifilm) by using ECL reagents (GE Healthcare, Buckinghamshire, UK) [41,43].

### 3.3. Oxygen Consumption Rate

The cellular oxygen consumption rate was measured by the 782 Oxygen Meter (Strathkelvin Instruments, Scotland, UK). The reaction was kept at 37 °C with a circulation water system. A total of 10^6^ cells were suspended in a 330-μL assay buffer (125 mM of sucrose, 65 mM of KCl, 2 mM of MgCl_2_ and 20 mM phosphate buffer, pH 7.2) and transferred into a closed chamber of the oxygen meter to measure the oxygen consumption rate of the cells. After the steady state was achieved, 1 μL of ethanol-purified digitonin (a final concentration of 0.0003%, Sigma-Aldrich) was added to the chamber to permeabilize the plasma membrane, which enabled direct exposure of the respiratory enzyme complexes to the following substrates. An aliquot of 10 μL succinate (1 M) (a final concentration of about 30 mM) was loaded into the chamber for 2 min to measure the succinate-supported oxygen consumption rates (nmol/min/10^6^ cells) [41]. Each experiment was performed in duplicate and repeated for 3 independent experiments (*N* = 3). The mean value and standard deviation were calculated for data presentation.

### 3.4. Lactate Concentration

A total of 10^6^ cells were seeded in 6-well culture plate and incubated for 24 h to achieve the steady state. They were then washed with PBS and replenished with 2 mL of fresh growth medium in 37 °C incubator for 4 h. Ten microliters of the medium was transferred to 96 well plate and mixed with the Lactate Reagent (Trinity Biotech plc., Bray, Ireland). Absorbance at 540 nm was measured on an ELISA reader (PowerWaveX 340), and the value was normalized by the cell number. Each experiment was performed in duplicate and repeated for 3 independent experiments (*N* = 3). The mean value and standard deviation were calculated for data presentation [41].

### 3.5. Intracellular ATP Content

The intracellular ATP content was measured by the ATP Bioluminescent Somatic Cell Assay Kit (Sigma-Aldrich) as described previously [41,46]. A total of 10^6^ cells were seeded on a 6-well for 24 h to achieve steady status. To release the intracellular ATP, cells were trypsinized and were re-suspended with 1 mL of growth medium, and then 50 μL of the suspended cells was mixed with 150 μL of Somatic Cell Releasing Reagent. Half of the mixture was subsequently transferred into a black OptiPlate-96F 96-well plate (Packard Biosciences, Perkin-Elmer) which contained 100 μL of ATP Assay Mixture. The luminescence intensity was measured by Victor^2^™ 1420 Multilabel Counter (PerkinElmer Life and Analytical Sciences). The luminescence intensity was normalized by the cell number. Each experiment was performed in duplicate for 3 independent experiments (*N* = 3) and the mean ± SD was used for data presentation.

### 3.6. Trans-Well Migration Activity

Cell trans-well migration was assayed by using 24-well culture plate and millicell hanging cell culture inserts. The bottom of the insert was covered with a piece of membrane with 8-μm pores (Millipore). A 400-μL of growth media containing DMEM plus 10% FBS was added in the 24-wells, and 5 × 10^4^ cells suspended in 150-μL of growth media containing DMEM plus 1% FBS were seeded in the insert. Followed by 24 h of incubation at 37 °C in a standard cell culture incubator, cells were removed from the upper surface of the membranes of the insert with a cotton swab. Cells that migrated to the lower surface were fixed with methanol for 20 min and stained with 0.2% (*w*/*v*) crystal violet for 1 h. Three random areas under a light microscope (40×) were selected to count the invaded cells and the average number was got (cells/field). Each experiment was performed in duplicate and repeated for 3 independent experiments (*N* =3). The mean value and standard deviation were calculated for data presentation [47].

### 3.7. Growth Kinetics

A total of 5000 cells suspended in 100 μL of growth media were seeded on a 96-well microplasty reader plates (Corning Glass Works, Corning, NY, USA). After incubating for 24 (reference point), 48, 72, or 96 h at 37 °C, an additional 10 μL of AlamarBlue™ reagent (Invitrogen) was added to the cells and incubated for 4 h. The fluorescence intensity was measured by the Victor^2^™ 1420 Multilabel Counter (Perkin-Elmer Life Sciences) on a plate reader at 538 nm (excitation) and 590 nm (emission). The methods for calculation of the cell growth kinetics were described previously by Larson *et al*. [48]. Each experiment was done in duplicate and repeated for 3 independent subcultures (*N* = 3). The mean value and standard deviation were calculated for data presentation.

### 3.8. Virus Infection to Set up TFAM Knockdown ESCC Cells

Small hairpin RNA (shRNA) constructs designed by the National RNAi Core Facility of Academia Sinica in Taiwan (http://www.rnai.genmed.sinica.edu.tw/index.asp) were employed to generate desired TFAM knockdown TE1 cells. Briefly, a vector derived from the pLKO.1 backbone and harbored a specific sh-oligonucleotide with sequence against TFAM was packaged into as lentiviral particles to infect TE1 cells. Then a shRNA would be transcribed in the TE1 cells to block the translation of TFAM. The pLKO.1 backbone contains an ampicillin resistant gene driven by a prokaryotic promoter and a puromycin resistant gene driven by hPGK eukaryotic promoter to act as selection markers, respectively. Two restriction sites, *Age*I and *Eco*RI, were designed for sh-oligonucleotide insertion, and this sh-oligonucleotide was triggered by the U6 eukaryotic promoter. The general structure of the inserted sh-oligonucleotide was of 5′-CCGG-a forward target sequence-CTCGAG (D-loop sequence) and a reverse target sequence-TTTTT-3′. Two target sequences against TFAM were designed as pLKO. 1-sh-TFAM(96) and pLKO.1-sh-TFAM(97), respectively. The forward target sequences of TFAM(96) and TFAM(97) are 5′-CGTTTATGTAGCTGAAAGATT-3′ and 5′-GCAGATTTAAAGAACAGCTAA-3′, respectively. For comparison, an empty vector (pLKO.1-NT, pLKO.1 backbone harboring a null-target sequence, 5′-TCAGTTAACCACTTTTT-3′, inserted into *Age*I and *EcoR*I sites) was set to act as the control vector.

Initially, the pLKO.1-sh-mtTFA(96), pLKO.1-sh-mtTFA(97) and pLKO.1-NT were transformed to DH5α strain of *E. coli* and cultured in argarose plates rinsed with an ampicillin solution (100 mg/L). Single bacterial clone was selected and cultured in liquid terrific broth medium containing tryptone (12 g/L), yeast extract (24 g/L), 100% glycerol (4 mL/L), KH_2_PO_4_ (2.31 g/L), K_2_HPO_4_ (12.54 g/L) and ampicillin (100 mg/L). The DNA of the cultured bacteria was extracted and sent for direct sequencing using the forward and reverse primers of LKO.1 shRNA/F: 5′-ACAAAATACGTGACGTAG-3′ and LKO.1 shRNA/R: 5′-CTGTTGCTATTATGTCTAC-3′, respectively. After confirming the sequences of pLKO.1-sh-mtTFA(96), pLKO.1-sh-mtTFA(97) and pLKO.1-NT, they were further packaged as lentiviral particles.

About 1.5 × 10^6^ of TE1 cells were suspended in 3 mL of growth medium and were seeded in a 6 cm culture plate. After 24 h of incubation, the growth medium was changed to a fresh one containing polybrene (8 μg/L) and sufficient amount of virus particles (2 multiplicity of infection, 2 MOI) were added for virus infection. Twenty-four hours later, the medium was changed to a new one containing puromycin (2 μg/mL) for clone selection.

After virus infection, 3 new clones were established. TE1 cells harboring pLKO.1-NT after 2 MOI of virus infection were named as TE1-NT. TE1 cells harboring pLKO.1-sh-TFAM(96) after 2 MOI of virus infection were named as TE1-sh-TFAM(96). TE1 cells harboring pLKO.1-sh-TFAM(97) after 2 MOI of virus infection were named as TE1-sh-TFAM(97).

### 3.9. Statistical Analysis

The continuous variables among different cell lines were compared using analysis of variance (ANOVA) and between 2 cell lines were compared using student *t*-test if appropriate. The difference was considered significant when *p* < 0.05. Statistical analysis was performed using the SPSS 12.0 software (SPSS Inc, Chicago, IL, USA).

## 4. Conclusions

In conclusion, our experimental results showed that a high copy number of mtDNA may contribute to the high bioenergetic function of mitochondria and further confer an advantage for tumor invasion in ESCC cell lines.

## Figures and Tables

**Table 1:** Comparison of the relative mtDNA copy number, bioenergetic function and invasion activity of seven esophageal squamous cell carcinoma (ESCC) cell lines.

	Cell lines							*p*-Value
	
Biological variables	48T	81T	146T	TE1	TE2	TE6	TE9	ANOVA
Relative mtDNA copy number (%) *	108.9 ± 43.8	79.4 ± 3.5	79.7 ± 7.9	240.7 ± 54.2	73.2 ± 14.0	98.0 ± 42.9	121.8 ± 26.0	<0.001
Relative mRNA expression level †								
ND1 (mtDNA encoded ND1 gene)	1.71 ± 0.29	0.74 ± 0.32	0.91 ± 0.30	2.80 ± 0.73	0.75 ± 0.20	0.53 ± 0.16	1.65 ± 0.59	<0.001
Oxygen consumption (nmol/min/10^6^ cells)	-	-	-	-	-	-	-	-
Succinate-supported	6.86 ± 0.56	10.03 ± 0.16	10.22 ± 1.10	11.21 ± 0.11	8.10 ± 0.69	10.52 ± 0.82	8.18 ± 0.41	<0.001
ATP content (fmol/cell)	8.10 ± 0.36	8.50 ± 0.40	5.57 ± 0.21	10.67 ± 0.51	7.17 ± 0.29	5.20 ± 0.10	6.50 ± 0.30	<0.001
Lactate concentration (mM)	4.02 ± 0.03	4.79 ± 0.10	4.06 ± 0.27	3.34 ± 0.05	3.58 ± 0.12	4.37 ± 0.18	6.29 ± 0.29	<0.001
Transwell migration (cells/field)	54.7 ± 2.3	41.7 ± 3.5	5.7 ± 1.5	223.0 ± 15.9	3.7 ± 1.5	4.0 ± 1.0	103.0 ± 12.3	<0.001

* The relative mtDNA copy number of 143B cell is defined as 1.00 (100%);

† The relative ND1 mRNA expression level of 143B cell is defined as 1.00.

**Table 2:** Comparison of the relative mtDNA copy number, bioenergetic function and invasion activity of the parental TE1, control TE1-NT and TFAM knockdown TE1-sh-TFAM(97) cell lines.

Biological variables	Cell line			*p*-Value, *t*-test	*p*-Value, *t*-test
	
	TE1	TE1-NT	TE1-sh-TFAM(97)	TE1 *vs*. TE1-NT	TE1-NT *vs*. TE1-sh-TFAM(97)
Relative mtDNA copy number (%) *	221.7 ± 9.8	227.8 ± 5.1	152.5 ± 13.4	0.403	0.001
Relative mRNA expression levels †	-	-	-	-	-
ND1 (mtDNA encoded ND1 gene)	3.12 ± 1.04	2.90 ± 1.13	1.70 ± 0.11	0.823	0.050
Oxygen consumption (nmol/min/10^6^ cells)	-	-	-	-	-
Succinate-supported	8.36 ± 0.94	8.66 ± 1.16	6.36 ± 1.07	0.747	0.065
ATP content (fmol/cell)	10.13 ± 0.67	9.90 ± 0.44	7.27 ± 0.76	0.642	0.007
Lactate concentration (mM)	3.39 ± 0.19	3.36 ± 0.12	4.31 ± 0.34	0.826	0.010
Trans-well migration (cells/field)	230.7 ± 9.6	224.7 ± 6.5	95.7 ± 7.2	0.428	<0.001

* The relative mtDNA copy number of 143B cells was defined as 100.0%;

† The relative ND1 mRNA expression level of 143B cells was defined as 1.00.
